# Reference Values, Determinants and Regression Equation for the Timed-Up and Go Test (TUG) in Healthy Asian Population Aged 21 to 85 Years

**DOI:** 10.3390/ijerph20095712

**Published:** 2023-05-03

**Authors:** Teck Chye Tan, Yan Y. Guo, Dilys J. Ho, Nur Aidah Binti Sanwari, Patricia H. Quek, Rachel S. Tan, Felicia S. Yap, Mingxing Yang, Meredith T. Yeung

**Affiliations:** 1 Physiotherapy, SingHealth Polyclinics, Singapore 150167, Singapore; 2 Singapore Institute of Technology, Singapore 138683, Singapore

**Keywords:** Timed-Up and Go (TUG), normative reference values, regression equation, healthy population, physical function

## Abstract

The “Timed-Up-and-Go” test (TUG) is a standard assessment tool for functional mobility as it assesses several functional components, including balance, gait, and lower-extremity strength. It has good reliability and validity and is cost-effective and safe, making it an ideal screening tool for falls in various populations, such as older adults or various conditions. However, TUG interpretation relies on comparisons against local normative reference values (NRV), which few studies established for the Asian or younger population. Hence, this study aims to: (1) establish the normative reference values NRV for the population aged 21 to 85 years; (2) determine demographic and anthropometric variables that influence the TUG results; and (3) establish the regression equation of the TUG. A prospective, convenience sampling cross-sectional study recruited subjects aged 21–85 from the community to complete two TUG trials in various parts of Singapore. Variables collected include gender, age, height (meters, m), weight (kilograms, kg), and hand grip strength (HGS) (kg). The intraclass correlation coefficient (ICC) and 95% confidence interval (95% CI) determined test-retest, intra- and inter-rater reliabilities. TUG and variables were analyzed with descriptive statistics and multiple linear regression. *p* < 0.05 was accepted as statistical significance. Further, 838 subjects (542 females, 296 males) completed the data collection. The mean TUG time was 9.16 s (95% CI 9.01–9.3). Slower TUG was observed with advanced age and female gender. Multiple linear regression analysis demonstrated that age, height, and weight were the best variables to predict TUG scores. The regression formula presented as: TUG (second) = 9.11 + 0.063 (Age, years)—3.19 (Height, meters) + 0.026 (Weight, Kilograms) (R^2^ = 0.374, *p* < 0.001). This study provided the TUG NRV and regression formula for healthy Asian adults aged 21 to 85. The information may provide a quick reference for the physical function to interpret assessment findings and guide decision-making in various health and healthcare settings.

## 1. Introduction

The Timed Up and Go test (TUG), modified from the “Get Up and Go” test [1], was designed to quantify functional mobility and lower extremity function in adults [2,3]. It is a safe, time-efficient, reliable, and cost-effective method to evaluate functional mobility in healthy individuals by assessing balance, gait, and lower-extremity strength [4,5,6]. This test records the duration an individual requires to rise from a seat, walk 3 m, turn around, walk back, and sit down on the seat. The equipment required to perform the test is readily available, making it easily reproduced outside the clinical setting.

The TUG is used as a case-finding tool to identify high falls-risk individuals, such as those with acute and chronic stroke [5], Parkinson’s disease [5,7], vestibular disorders [8], and spinal injuries [5]. With high sensitivity and specificity as a predictor of mobility status and falls [9], it is also used extensively in fall screening [10], wherein high fall risk and low functional mobility are known indicators of mortality in older adults [11]. It is thus widely used for fall-risk assessments, goal-setting and as a functional outcome measure by clinicians worldwide [12]. The TUG has been proven to have high reliability and validity in multiple studies [5,12,13,14,15,16], reporting inter-rater reliability of 0.99 and excellent validity compared to Berg’s Balance Scale (r = −0.81) or the Barthel Index (r = −0.78) [3].

However, the interpretation of TUG results is dependent on comparisons against reliable normative reference values (NRV) [16], which are data that “characterize[s] what is usual in a defined population at a specific point or time” [17]. Therefore, it is recommended that comparisons be made against local population data [18], as TUG NRV have been shown to differ across regions. For example, a meta-analysis established that the mean time taken to complete the TUG by adults over age 60 was 8.74 s (s) in Asia, compared to 10.17 s in Europe [9]. Likewise, NRV in community-dwelling older adults in Spain showed a mean TUG of 8.02 s [Standard Deviation (SD = 2.68)] and 8.77 s (SD = 3.13) for males aged 70–74 years and 75–79 years, respectively [19]. This also differed from Thai males aged 70–79, who took a mean of 10.2 s (SD = 1.8) to complete the TUG [20]. Besides geographical differences, TUG has also been found to differ between age groups. Older adults have performed slower with the TUG; an Irish study reported that individuals aged the 50s took a mean of 7.9 s (SD = 1.8) to complete the TUG, as compared to those aged 70s required 10.0 s (SD = 5.1) [16]. This difference is associated with age-related degeneration, such as reduced reaction times and overall strength [21]. Additionally, it has been reported that females took longer to complete the TUG than males [18] due to physiological differences and performance between genders [22]. Recent studies exploring the relationship between TUG and gender have concluded that advanced age and female gender yield statistically worse TUG results [15,18,23]. Hence, ethnicity-, gender-, and age-specific results for the TUG are necessary.

Slower TUG has also been associated with higher body mass index (BMI) [6,24]. BMI is an indirect measure of obesity and has been associated with reduced functional mobility in individuals [24] due to biological, psychosocial, and environmental factors [25]. Previous studies show that a higher BMI is primarily associated with a shorter chronic disease-free life and increased fall prevalence [26,27,28,29]. Kim et al. (2016) postulated that a U-shaped correlation exists between BMI and falls, where underweight and obese groups risk more falls than normal BMI [30]. Therefore, we can hypothesize that a similar U-shaped correlation exists between TUG and BMI. Due to different inclusion criteria and demographics, this cannot be concluded from existing studies.

Reduced upper limb muscle strength, specifically poor handgrip strength (HGS), is correlated with increased mortality [31] and slower TUG [14]. For reasons such as that upper limb strength is more closely related to activities of daily living (ADLs) that require upper body strength and mobility, these tasks are essential for maintaining independence and quality of life in older adults [32]. Furthermore, upper limb strength may be a more sensitive indicator of age-related decline in physical function than lower limb strength since upper limb muscle mass and strength decline faster with age than lower limb muscle mass and strength [33]. Lastly, the measurement of upper limb strength is a more practical and feasible measure of frailty in clinical settings, as the assessment of lower limb strength often requires more sophisticated instruments and trained personnel, whereas upper limb strength can be assessed using simple, low-cost tools such as handheld dynamometers or grip strength tests [34]. The grip strength test has established its predictive power on both short- and long-term mortality [33,34,35]. A Singapore study [36] showed that HGS is a “strong and independent predictor of short-term mortality in an elderly Chinese community-dwelling population in Singapore”. Hence, HGS and TUG are standard screening tests for sarcopenia and muscle strength assessment, yet little effort has been attempted to corroborate such correlations locally.

Judgement of the individual test results should be compared with that of a relevant population, requiring the availability of norm references for the particular population [37]. Few studies have explored the NRV of TUG in Singapore, with most focusing on adults aged 50 and above [38]. The NRV for young adults in Singapore are lacking but may be required by care providers for health assessment and goal-setting in younger patients. Therefore, establishing NRV for a younger population would prove helpful [6]. Furthermore, observing correlations between physical variables and TUG may identify risk factors in individuals and the population, thus allowing for implementing preventative and restorative strategies in numerous health conditions. Consequently, this study aims to establish the NRV and regression formula for TUG in healthy Singaporean adults and to explore the relationships between age, gender, height, weight, BMI, and HGS with TUG.

## 2. Material & Methods

### 2.1. Study Design and Recruitment Process

The University—Institutional Review Board approved this convenience sampling cross-sectional study (Project Number: 2019100), which took place between August 2019 and March 2021 at various community centers in Singapore. Informed written consent was obtained from each subject before commencing data collection.

### 2.2. Subjects

Convenience sampling was used to obtain a representative sample of the population, fulfilling a minimum quota of 30 subjects per gender per age group. Subjects recruitment took place at various community centers in Singapore with physical recruitment booths set up. We recruited community-dwelling, healthy Singaporeans who visited community centers on the occasions where recruitments took place. No pre-contact or -schedule of any subjects was necessary. All subjects completed a screening questionnaire to determine suitability. Inclusion criteria were community ambulant Singapore residents between 21 and 85 years old. Subjects were excluded if they had: any visual, auditory, or neuromuscular impairments; psychiatric or psychological disorders which could affect adherence to or comprehension of instructions; recent musculoskeletal injuries and/or surgery affecting gait; any acute and/or chronic respiratory or cardiac disease or cancer in the last six months; resting heart rate (HR) > 100 beats per minute (bpm) or <50 bpm; resting systolic blood pressure (SBP) > 150 or <90 mmHg, diastolic blood pressure (DBP) > 100 or <50 mmHg; oxygen saturation (SpO_2_) < 95% at rest. Baseline measurements included height, weight, BP, HR, SpO_2_, and HGS. All data collection was completed within a single occasion, and no follow-up was necessary.

### 2.3. Timed Up and Go Test (TUG)

This study used a single-model wooden armchair with a backrest, a seat height of 0.46 m (m), and an armrest height of 0.67 m. The TUG walkway was marked with tape 3 m away from the front edge of the chair. Subjects were seated in the chair with their backs touching the backrest, arms on the armrests, and briefed with the original standardized TUG instructions [3,4]. Abided by the standard guidelines [3,39], the following instructions: “On the word ‘go’, you will stand up, walk to the line on the floor, turn around and walk back to the chair and sit down. Walk at your regular pace”. The test’s timing began with the word “go” and ended when the subject was seated. A demonstration by the investigators was given before a practice trial was conducted. All subjects completed two trials of TUG, with the average time used for data analysis. No breaks were given between both timed trials unless requested by the subject. The researchers measured the time taken manually with a stopwatch and were familiar with the TUG protocol. Data collection on 30 subjects was conducted to establish the test-retest, intra-, and inter-rater reliability of TUG between the investigators.

### 2.4. Grip Strength Test

The grip strength test followed an established protocol [40] and standardized instructions [41], with an intra-class correlation coefficient (ICC) of 0.95 reported. The Jamar^®^ Plus+ Digital Hand Dynamometer was used to measure the HGS of the subjects’ dominant hand. HGS was measured in kilograms (kg) with an accuracy of 0.1 kg. The dynamometer was set to 3.8 cm at the second handle position. Subjects seated with back unsupported, hips and knees at approximately 90 degrees, positioned their dominant arm with “shoulders adducted and neutrally rotated, elbow flexed at 90 degrees, forearm in a neutral rotation, and the wrist between 0 and 30 degrees extension and between 0 and 15 degrees ulnar deviation” [40,41]. The researchers were trained and familiar with the grip strength test protocol. A demonstration by the investigator was given before a practice trial was conducted. All subjects completed two trials of the HGS with a 5-min rest interval between each trial; the highest reading was used for data analysis.

### 2.5. Statistical Analysis

GraphPad Prism Version 8.4.3 (686) (GraphPad Software, San Diego, CA, USA) was used for the statistical analysis. Descriptive statistics reported the characteristics of all participants with mean (SD) and 95% confidence interval (95% CI). Interclass correlation coefficient (ICC) determined the reliabilities of inter- and intra-raters and the consistency of the test when repeated over two occasions with the same group of subjects (test-retest). Repeated measures, a two-way random-effects model, and absolute agreement ICC with 95% CI were also used to assess reliability. Using the 95% CI of the ICC estimate, the reliability value was ranked accordingly: ICC < 0.5 (poor), 0.5 ≤ ICC < 0.75 (moderate), 0.75 ≤ ICC <0.9 (good), ICC ≥ 0.9 (excellent) [42]. One-way analysis of variance (ANOVA) with 95% CI identified the upper bound and cutoff values to establish NRV for healthy adult Singaporeans across different age groups, split as 21–39 years, 40–59 years, 60–69 years, and ≥70 years old. The Independent-Samples *t*-Test analyzed TUG between genders, while Tukey’s Honest Significant Difference (HSD) range test was used between age groups. Pearson product correlation coefficient (r) and multiple linear regression explored the relationships between age, gender, height, weight, BMI, HGS, and TUG. Statistical significance was accepted when *p* < 0.05.

## 3. Results

### 3.1. Reliability

Table 1 presents the test-retest [ICC (SD) = 0.879 (0.34); 95% CI (0.838 to 0.911); SEM = 0.12] and inter-rater [ICC = 0.953 (1.03); 95% CI (0.919 to 0.975); SEM = 0.22] reliabilities. The intra-rater data of the three investigators [Rater 1, ICC = 0.804 (0.68); 95% CI (0.681 to 0.891); Rater 2, ICC = 0.858 (0.66); 95% CI (0.772 to 0.921); Rater 3, ICC = 0.821 (0.69); 95% CI (0.701 to 0.901)].

### 3.2. Subject Characteristics and TUG

**Figure 1:**
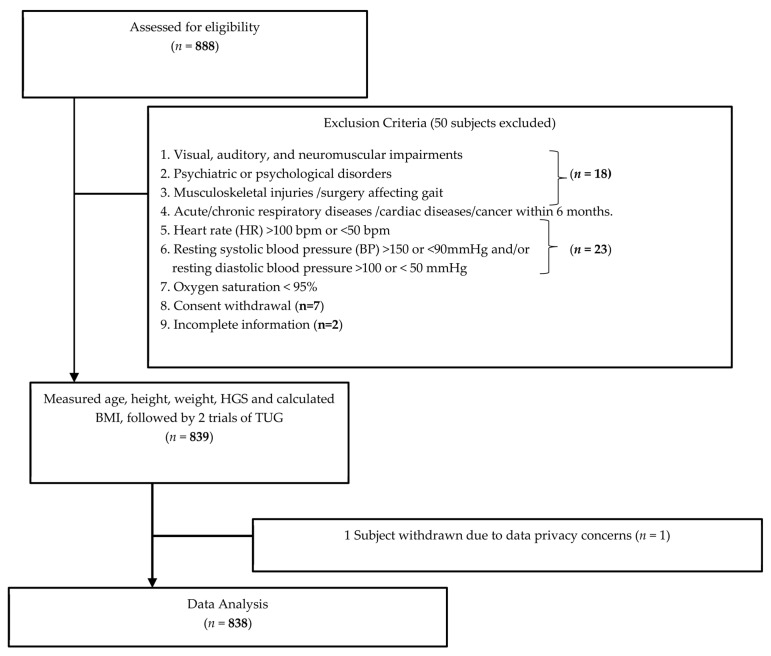
Subject recruitment process.

Initially 888 subjects were recruited for assessment (Figure 1). Fifty subjects were excluded due to consent withdrawal (*n* = 7), past medical history (*n* = 18), baseline vitals (*n* = 23), and incomplete information (*n* = 2). Therefore, 838 subjects were included for data analysis.

**Figure 2:**
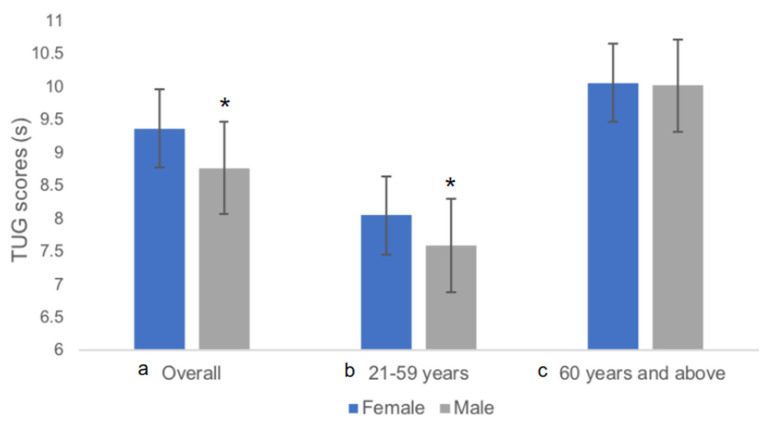
Comparison of TUG between female and male genders in the (**a**) overall, (**b**) 21–59 years subgroup, and (**c**) ≥60 years subgroup. * *p* < 0.001.

Table 2 presents the demographics of all subjects and the NRV of the TUG, and the one-way ANOVA comparing TUG between the age-stratified groups with Tukey’s HSD. The mean age (SD) of subjects was 56.0 (19.1) years. Further, 64.7% of the subjects were female, while 59.4% were ≥60 years old. The population mean TUG time was 9.16 s [SD 2.12, 95% CI (9.01 to 9.30)], with the fastest at 4.52 s and the slowest at 21.20 s. Significantly, there was a corresponding increase in TUG with an increase in the age group. Results indicate that subjects over 70 years have significantly slower TUG than adults in all other age groups (*p* < 0.001). Overall, males completed TUG in 8.77 s [SD 2.05, 95% CI (8.53 to 9.00)] compared to 9.37 s [SD 9.37, 95% CI (9.19 to 9.55)] in females (95% CI: −0.90 to −0.30, *p* < 0.001), and TUG were only significantly different between genders in the younger adult group (*p* < 0.001) but not with the older adult group (*p* = 0.8462). Figure 2 illustrates the TUG differences between females and males in (a) overall, (b) 21–59 years subgroup, and (c) ≥60 years subgroup.

### 3.3. Relationship between TUG and Variables

The relationships between TUG, demographics, and anthropometric variables are presented in Table 3. Notably, only age (r = 0.597; *p* < 0.001), height (r = −0.327; *p* < 0.001), and weight (r = −0.074; *p* =0.325) attained moderate to weak correlations, respectively, with TUG with statistical significance. The weak to negligible correlation [43] of overall BMI (r = 0.133; *p* < 0.001), together with the breakdown analyses [BMI: Underweight (r = 0.1141; *p* < 0.445); BMI: Normal to Obese (r = 0.155; *p* < 0.001)], remained inconclusive. HGS and TUG could not establish statistical significance.

Table 4 depicts the multiple linear regression analysis, using age, height, and weight as the dependent variables, established the regression formula for TUG (R^2^ = 0.374, *p* < 0.001) in seconds, with age in years, height in meters, and weight in kilograms:

TUG (second) = 9.11 + 0.063 (Age, years)—3.19 (Height, meters) + 0.026 (Weight, Kilograms)

## 4. Discussion

This study is one of the few to report the TUG NRV and reference equation in the Asian population. With an extensive age range of 21 to 85 years old, the reference formula explains 37.4% of the variance (*p* < 0.001). Similar to the rest of the Asia-pacific regions, Singapore faces the challenge of an ageing population. Establishing the TUG NRV provides a benchmark to screen for the risk of sarcopenia, frailty, and falls. There was a statistically significant increase in TUG across all age cohorts. The mean (SD) TUG for our subjects in the 60–69 year group was 9.48 (SD = 1.62) s, and the ≥70-year group took 10.68 (SD = 2.22) s, both of which are higher than the NRV from a Japanese study [15] and meta-analysis reports [4,9] which analyzed mainly Caucasian studies. One possible reason could be that Caucasians broadly exhibit anthropometric differences compared to Asians, particularly Singaporeans. On the contrary, younger Singaporean adults (between 21–59 years) completed the TUG faster than their counterparts from the United States [6] but remained slower than the Japanese subjects [15] (50–59 years). However, only one study by Kear et al. (2017) reported the NRV of TUG for ages 20 to 59 years [6]. It is premature to conclude the TUG trend in the younger adult population, primarily because Kear et al. (2017) subjects were recruited from a clinic, unlike the healthy adults in this report.

Age was the most substantial correlated factor to TUG (r = 0.597, *p* < 0.001), even higher than previous reports from India (r = 0.417) [44] and Spain (r = 0.25) [23]. However, as reflected in previous studies [4,6,9], TUG only increased with age between broadly-stratified younger (21–59 years) and older (≥60 years) groups. Further analysis between age groups showed significance only when comparing age groups twenty years apart in the younger adult population. Similar to Kear et al.’s study [6], TUG was not different between each sub-decade of age below 60 years. This means that TUG was not vastly different in the younger adult population, unlike the differences observed between the 60s [9.48 (SD = 1.66) s] and ≥70s [10.68 (SD = 2.12) s] age groups. The implication is that fall risk increases at a higher rate once an individual is 60 years or older. This finding is corroborated by a local Singapore study [45], which stated that the mean age of fallers in the country was above 60. Similarly, a Netherlands study [46] previously reported that fall injury increased exponentially beyond age 70, and a TUG longer than or equal to a cutoff of 10.85 s predicts sarcopenia with a sensitivity of 67% and specificity of 88.7%—a cutoff value well-dividing the NRV between the 60s vs. those ≥ 70s in this current study.

Generally, females took longer to complete the TUG than males, consistent with previous studies [18,23,46,47]. Female subjects were shorter and weighed less than the males, possibly contributing to slower TUG due to shorter stride length, reduced gait speed, and decreased muscle mass [18,23]. Gender seems to play a more prominent role in poorer performance in the older adult population. Females show an acceleration in muscle mass loss after 55 years, and there is a more significant correlation between strength and functional mobility in females compared to males, indicating that they function closer to their strength-related limits [48]. This results in TUG being more affected in females than in males. These findings and postulation are consistent with reports that falling is more prevalent in older females [45]. Despite this, gender was not a significant variable for the regression equation. Unfortunately, the current study data cannot elucidate this phenomenon.

Height (r = 0.327, *p* < 0.001) is found to be the second most important variable influencing the TUG in the current study; while on the contrary, weight (r = −0.074, *p* < 0.0325) only correlates negligibly to TUG, differing from previous studies [23,44] that demonstrated both as influencing variables. Comparably, a very weak positive correlation was observed between TUG and BMI (r = 0.133, *p* < 0.001), which is consistent with previous studies [6,24,36] despite different mean age groups reported. This trend suggests that the TUG and BMI correlation is possibly independent of age. However, the current correlation between BMI and TUG was weak and could not contribute significantly to the multiple linear regression analysis. This could be because BMI is not an accurate indicator of muscle mass, especially in younger, fit adults [49]. Muscular obesity results from a higher muscle mass, contributing to the BMI cutoff value. Additionally, BMI considers both height and weight. The almost negligible correlations between weight and TUG in the current study could have influenced the BMI’s usefulness in result predictions. Our findings differed from a previous study that showed no correlation with height in institutionalized and community-dwelling older females [13]. Nevertheless, height is not a modifiable factor and cannot be targeted in interventions. Remarkably, the results of HGS did not demonstrate any correlation with TUG in this study (r = 0.023; *p* = 0.744). However, HGS has value as an independent predictor of frailty, with which TUG is also positively correlated [38].

TUG is predictive for sarcopenia [50], frailty [3,38], and falls [8,12,36,51]. Several studies have suggested different TUG times as cutoffs for falls and frailty, ranging from <12 s to 13.5 s [12,18]. However, these values were derived from mostly non-Asian demographics, which may not be accurate for judging the individual test results when used in our population [37]. A Singapore study previously reported a TUG range of 10.5 s to 12.3 s in the robust and pre-frail groups, respectively [38], while another study reported that fall risk increases exponentially over 70 years old [51]. These values coincide with the NRV of TUG in this study’s ≥ 70 years group. By extrapolation, we can deduce that individuals over 70 and TUG > 10.5 s are at risk of falls. It may be beneficial for further studies to explore the correlation between fall risk and TUG, using this data as a benchmark to determine frailty and at-risk individuals in our local population.

One of the limitations of this study was the disproportionate distribution of subjects, which could not be prevented through random sampling with minimum quota allocation. This study included 838 subjects aged 21–85, with more female subjects (64.7%), resulting in a potential gender selection bias. Additionally, the study subjects were recruited from those who physically visited the various community centers in Singapore, leading to another slight bias regarding the general physical fitness and well-being of these subjects. One can naturally assume that only those with sufficient physical fitness/threshold would visit such community centers frequently. Indirectly, this explained the relatively small excluded sample (*n* = 50) despite the notably sufficient sample size of 888 initially recruited. Nonetheless, results from this study are consistent with prior studies, indicating that females and individuals of advancing age tend to take longer to complete the TUG than males.

## 5. Conclusions

This study described the NRV for TUG in a healthy adult population in Singapore with an extended age range from 21 to 85. Older age and female gender were correlated with slower TUG, with age being the most significant predictor. Our regression equation or age-stratified NRV may thus be most beneficial in establishing benchmarks for health assessment, treatment outcomes, and goal-setting for physical function. Determining the variables most associated with slower TUG and, therefore, increased fall risk enables early identification of at-risk individuals. Early and targeted intervention, especially for modifiable risk factors such as obesity, can subsequently be carried out, thus leading to fewer falls in the community.

## Figures and Tables

**Table 1:** Test-retest, inter-rater and intra-rater reliabilities.

	ICC (SD)	95% CI	SEM
Test-retest	0.879 (0.34)	0.838 to 0.911	0.12
Inter-rater	0.953 (1.03)	0.919 to 0.975	0.22
Intra-rater			
Rater 1	0.804 (0.68)	0.681 to 0.891	0.30
Rater 2	0.858 (0.66)	0.772 to 0.921	0.25
Rater 3	0.821 (0.69)	0.701 to 0.901	0.29

Note. ICC = Intraclass Correlation Coefficient; SD = standard deviation; 95% CI = confidence interval; SEM = standard error of measurement.

**Table 2:** Demographics of subjects: age, gender, height, weight, body-mass index, and normative reference values (NRV) and One-way ANOVA comparing TUG times between age-group and Tukey’s Honest Significant Difference (HSD) range test for between-group differences.

Age (Years)					Height (m)		Weight (kg)		BMI (kg/m^2^)		Normative Reference Values													
Decades	Gender	n	Mean	SD	Mean	SD	Mean	SD	Mean	SD	Mean	SD	Median	Interquartile Range	Min	Max	95% CI Lower Bound	95% CI Upper Bound	10th Percentile	90th Percentile	Tukey’s HSD Range Test Compares Group Differences	Mean Difference in TUG Time	95% CI (Mean Difference)	*p*-Value
21–85	Total	838	56.0	19.1	1.59	0.08	61.00	12.45	23.90	4.03	9.16	2.12	8.91	7.70–10.31	4.52	21.20	9.01	9.30	11.91	6.81				
21–39	Total	197	24.4	3.4	1.67	0.08	66.40	14.32	23.72	4.30	7.20	1.24	7.19	6.27–8.02	4.52	10.35	7.02	7.37	8.84	5.52				
																					40–59	−1.54	−2.02 to −1.07	<0.001
																					60–69	−2.29	−2.70 to −1.88	<0.001
																					70–85	−3.49	−3.91 to −3.07	<0.001
	Females	79	24.6	4.4	1.60	0.06	59.30	14.33	22.93	4.90	7.13	1.20	7.17	6.24–7.84	4.95	10.35	6.86	7.40	8.55	5.57				
	Males	118	24.2	2.6	1.71	0.06	71.20	12.21	24.25	3.77	7.24	1.27	7.26	6.32–8.22	4.52	9.94	7.01	7.47	8.87	5.48				
40–59	Total	143	53.2	5.3	1.59	0.08	61.60	11.60	24.38	4.10	8.74	1.33	8.60	7.81–9.50	5.66	14.48	8.52	8.96	10.32	7.33				
																					60–69	−0.74	−1.20 to −0.29	<0.001
																					70–85	−1.95	−2.41 to −1.48	<0.001
	Females	108	53.0	5.4	1.56	0.05	59.50	10.08	24.50	3.85	8.73	1.36	8.26	7.62–8.70	5.66	9.56	8.46	8.98	9.09	7.19				
	Males	35	53.8	4.7	1.69	0.07	68.20	13.51	24.00	4.56	8.78	1.25	10.05	9.83–10.94	9.56	14.48	8.35	9.21	11.88	9.70				
60–69	Total	261	65.2	2.8	1.57	0.07	59.60	11.79	24.20	4.15	9.48	1.66	9.33	8.42–10.36	6.53	15.93	9.29	9.68	11.46	7.51				
																					70–85	−1.2	−1.59 to −0.81	<0.001
	Females	194	65.2	2.8	1.54	0.05	57.20	10.75	24.10	4.16	8.42	1.59	9.22	8.39–10.33	6.53	15.93	9.19	9.64	11.21	7.45				
	Males	67	65.1	3.0	1.65	0.06	66.40	12.09	24.50	4.15	9.68	1.69	9.68	8.53–10.43	6.84	14.71	9.27	10.09	11.71	7.58				
≥70	Total	237	73.7	3.2	1.56	0.08	57.60	10.43	23.60	3.60	10.68	2.12	8.91	7.70–10.30	6.99	21.20	9.01	9.30	11.91	6.81				
	Females	161	73.6	3.1	1.52	0.05	55.00	9.39	23.70	3.82	10.85	2.31	10.94	9.30–11.98	6.99	21.20	10.49	11.21	13.46	8.40				
	Males	76	74.1	3.3	1.64	0.07	63.20	10.07	23.30	3.09	10.33	1.97	10.04	8.83–11.83	7.03	17.02	9.89	10.78	12.72	8.05				

*n* = number of participants; m = meters; kg = kilograms; BMI = body-mass index; SD = standard deviation; min = minimum; max = maximum; 95% CI = 95% confidence interval.

**Table 3:** Relationship between TUG and variables.

Independent Variable	Pearson’s r	*p*-Value
Age	0.597	<0.001 *
Gender	−0.120	0.331
BMI	0.133	<0.001 *
Underweight	−0.114	0.445
Normal to Obese	0.155	<0.001 *
Height	−0.327	<0.001 *
Weight	−0.074	0.0325
HGS	0.023	0.744

* *p* < 0.05 represents a significant value.

**Table 4:** Linear regression model for predicting TUG.

	Coefficient	Standard Error	*p*-Value	95% Confidence Interval
**R^2^ = 0.374**	9.11	1.366	<0.001	6.43 to 11.79
Age	0.063	0.003	<0.001	0.057 to 0.070
Height	−3.19	0.881	<0.001	−4.92 to −1.46
Weight	0.026	0.006	<0.001	0.015 to 0.037
BMI	0.142	0.130	=0.273	−0.112 to 0.396

## Data Availability

The data sets generated and/or analysed during the current study are available from MTY upon reasonable request.
